# Supplementary material for: Surf smelt accelerate usage of endogenous energy reserves under climate change
Source: PLoS One. 2022 Jun 27;17(6):e0270491. doi: 10.1371/journal.pone.0270491 (PMC9236230; doi:10.1371/journal.pone.0270491)
Supplement: S1 Table — Average seawater temperature, pH, pCO2, and CT for the embryo heartrate experiment. Data are shown as time-averaged means ± 1 SD of (n) measurements. pH and temperature were measured daily, while CT was measured 3 times per week. pCO2 was derived from temperature, pH, and CT measurements. (PDF) [file pone.0270491.s001.pdf]

Conditions (C_T + °C)	*In-Situ* Measurements pH (NBS Scale)	Temperature (°C)	Discrete Samples pCO_2 (µatm)	C_T (µmol kg*SW^{-1})	Salinity
ambient +13	7.86 ± 0.01 (9)	13.56 ± 0.26 (9)	839.89 ± 1.03 (3)	2020.46 ± 7.10 (3)	30.3 ± 0.5 (3)
ambient +15	7.84 ± 0.01 (9)	15.04 ± 0.08 (9)	884.78 ± 4.89 (3)	2015.65 ± 8.51 (3)	30.1 ± 0.2 (3)
ambient +18	7.84 ± 0.02 (9)	17.98 ± 0.19 (9)	940.38 ± 55.21 (3)	2011.86 ± 3.41 (3)	30.1 ± 0.2 (3)
elevated+13	7.43 ± 0.04 (9)	14.10 ± 0.32 (9)	2243.73 ± 203.91 (3)	2066.24 ± 1.87 (3)	30.1 ± 0.2 (3)
elevated+15	7.42 ± 0.04 (9)	15.10 ± 0.19 (9)	2342.09 ± 239.96 (3)	2067.92 ± 16.48 (3)	30.3 ± 0.2 (3)
elevated+18	7.46 ± 0.04 (9)	17.98 ± 0.07 (9)	2209.26 ± 135.67 (3)	2057.98 ± 8.30 (3)	30.1 ± 0.2 (3)
