# Supplementary figures and images for: Surf smelt accelerate usage of endogenous energy reserves under climate change
Source: PLoS One. 2022 Jun 27;17(6):e0270491. doi: 10.1371/journal.pone.0270491 (PMC9236230; doi:10.1371/journal.pone.0270491)

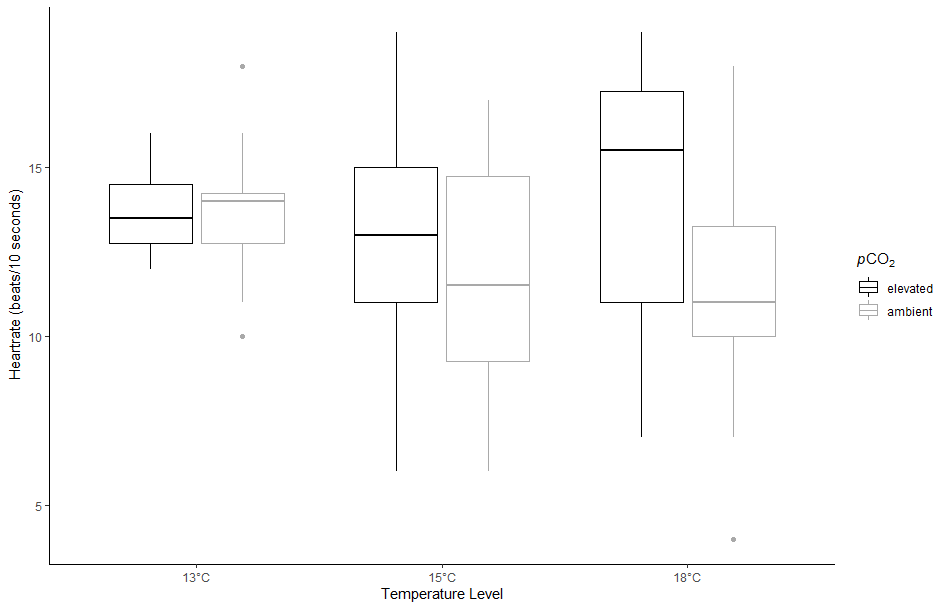

Supplement: S1 Fig — The number of heart beats from embryos (n = 96) per ten seconds from each treatment. Whiskers extend from the upper and lower quartiles to 1.5 times the interquartile range. Data outside of this range are shown as points. Neither temperature nor pCO2 were significant predictors of heartrate. (TIF) [file pone.0270491.s002.tif]
