# Supplementary material for: Surf smelt accelerate usage of endogenous energy reserves under climate change
Source: PLoS One. 2022 Jun 27;17(6):e0270491. doi: 10.1371/journal.pone.0270491 (PMC9236230; doi:10.1371/journal.pone.0270491)

PLoS One review
Russell et al
This paper poses that beach spawning forage fish, smelt, may be affected by increasing temperature and seawater CO_2 and possible interactions between these variables. The question is interesting, but tricky. I've provided some additional things for the authors to consider below. The accelerated use of yolk at high temperatures is clear but not unexpected. The effects of CO_2 and interactions with temperature are minimal/none. The paper is generally well-written.

1. The eggs are found buried in sediment on the beach and its unclear how much time is spent immersed in seawater. At lower tides, the eggs are in a moist environment that may heat up due to sun exposure. I imagine the CO_2 levels in the sediment increase at this point relative to that found in seawater. No measurements of pH or CO_2 in the sand were made. If CO_2 is higher in seawater than in the emmersed sand, then one would expect high CO_2 to cooccur with lower (ambient) temperatures. However, the only interaction between CO_2 and temperature found was at high temperature and high CO_2 (but see point 2).
2. Heart rate was highly variable but not significantly different across temperatures. The high Temp/high CO_2 treatment was significantly lower but the error bars are enormous and completely overlap with the other treatments. So that result is not compelling. The text states that heart rate increased at high temp/ambient CO_2, but that is not significant and so should be changed (no effect).
3. The text makes the case that the reduced heart rate may have resulted from CO_2/pH effects on oxygen binding in the blood. However, this would only be important if the embryos were near maximum metabolic rate for the available oxygen (i.e. near the Pcrit for whatever their oxygen consumption rate was). Aerobic scope (difference between max and rest metabolic rates) is largely unknown for eggs. However, P_{crits} for fish eggs below 50% saturation have been reported. In that case, any reduction in oxygen transport would not translate into a reduced metabolic rate under the oxic experimental conditions. Something to ponder.
4. Heart would be expected to increase with metabolic rate, which nearly always increases with temperature. An increased metabolic rate is consistent with greater consumption of yolk and fat stores at high temperature. Its unclear why heart rate wouldn't increase at high temperature except that its so variable as to be undetectable.
5. There is a small, but likely significant, "experiment" effect on the CO_2 levels achieved (see figure below). The embryo heart rate experiment had the highest PCO_2 levels achieved of any experiment. Its not possible to say whether than influenced the outcome relative to the slightly lower values in the other two experiments.
6. As pointed out by the editor, the paper discusses PCO_2 in the introduction and frames hypotheses about the effects of PCO_2 on biology. However, the authors instead perform statistical analyses on total carbon. As stated on line 124: "*While pCO₂ is the parameter most commonly used to represent the suite of changes associated with OA, and is the parameter actually manipulated in many systems, CT is the control variable in this system.*" It is further stated that PCO_2 is temperature sensitive and this is why CT was used instead. However, both variables are temperature sensitive. In fact, what changes with temperature is the solubility of CO_2. If a given amount of CO_2 is dissolved in seawater, both total carbon and carbon dioxide pressure will change and this change will be different at different temperatures because of changing solubility with temperature. Cold water will hold more of the CO_2 (thus higher CT) and the dissolved CO_2 will exert less pressure. This is seen clearly in a graphical representation of the data (below), especially within the "ambient" treatment. So ultimately it doesn't matter which is stated throughout the text…the treatment has high total CO_2 *and* high PCO_2 relative to the ambient control. However, because the range of values is different for each variable, the statistical analyses could be influenced. For example, if the PCO_2 doubles, the pH will decrease by 0.3 units and total CO_2 increases by only a small fraction because of the large amount of CO_2 already

dissolved in seawater. Thus analyzing the interacting effects of CT and temperature may provide less clear results than analyzing the interacting effects of PCO_2 and temperature…even though they are two sides of the same coin.   Because CO_2 was added at a particular rate, rather than a particular amount or to achieve a particular PCO_2, pH or TC, the various carbonate parameters all change with temperature in interrelated ways and the different experiments have different values.

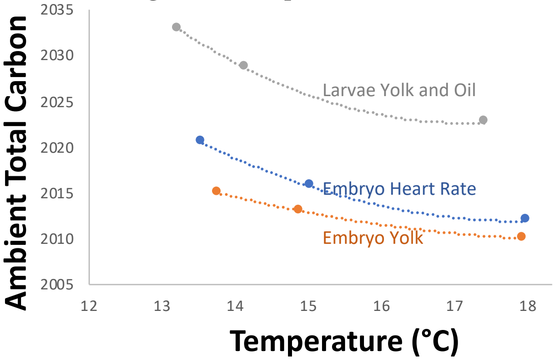

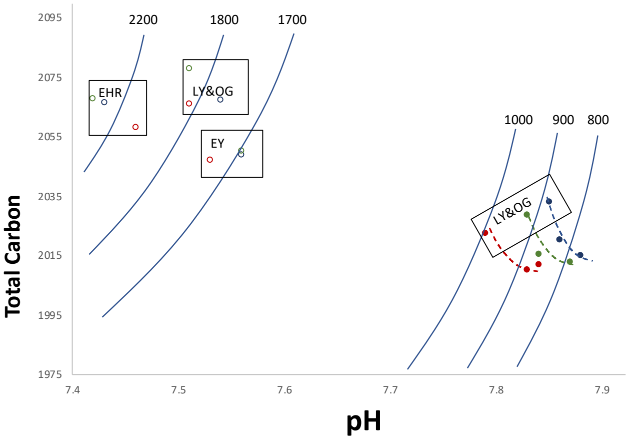

Supplement: Attachment — Submitted filename: Russell et al smelt CO2 temp PLoS One review.pdf [file pone.0270491.s005.pdf]
