# Supplementary material for: Surf smelt accelerate usage of endogenous energy reserves under climate change
Source: PLoS One. 2022 Jun 27;17(6):e0270491. doi: 10.1371/journal.pone.0270491 (PMC9236230; doi:10.1371/journal.pone.0270491)
Supplement: Attachment — Submitted filename: Review for.docx [file pone.0270491.s003.docx]

Review for “Surf smelt accelerate usage of endogenous energy reserves under climate change”

This is an interesting manuscript in which the short-term effects of high temperature and high pCO2 exposure on energy reserves of embryos and larvae of surf smelt are described. I have several questions regarding the experimental design and a few remarks for the results section. The discussion is much too long and often discusses topics that are only distantly related to what was actually measured in this study. The authors describe what other studies found but often miss to connect it to their own findings.

Abstract

Line 12-14: One can also argue that because surf smelt are beach spawners, they are exceptionally robust to environmental stress

Line 18:19: Why are you specific with the temperature used but not the pCO2 level? At least give an approximation what “ambient” and what “elevated” means.

Line 26: “CT” has not been explained before use

Line 27: “may impact”. It is more informative if you give a direction. Is it negative or positive impact?

Introduction

Line 107-109: This sentence is repetitive to the previous sentence

Methodology

Line 130: Refer to Table 1.

Line 135-136: Figure 1 is not needed. If you want to show it somewhere put it in the supplementary material

Line 142: Were the embryos shock exposed to the treatment conditions or was there a gradual increase/decrease in water temperature and pCO2?

Line 145-150: Why did you not use the embryos from your embryo experiment to be further studied as larvae? There might be cascading effects from embryo to larva that you missed because of your experimental design which I see as a drawback for your results

Line 172-174: I don’t understand what is meant. Were the temperature treatments not run at the same time?

Line 185-188: What was done to maintain treatment conditions during measurements of heart rates? How long after removal of the embryos from their tanks did it take to start recording the heart rate videos?

Results

Line 206-227: I find this a very weird way of showing the treatment conditions. It gives the impression that you did not exactly know what the treatment conditions were. E.g. line 208: “temperatures remained consistent throughout the duration of the experiment”. If your experiment was planned to use stable temperatures, this is a given. I consider this unnecessary to take up so much space in your manuscript. Referring to Table 1 in your methods section would simply be enough.

Line 211-214: Why is the treatment then called 15C instead of 16C if the water temperature was 16C and not 15C?

Line 213: “summer warming elevated the seawater temperature in the ambient temperature treatment” by how much? For how long?

Line 235-237: Figure 2 does not show this result. I suggest to use a glmer to account for the ratio which cannot be above 1 or below 0. Also I would prefer showing the error as 95% intervals and offsetting the CT data for clarity of the error bars. Maybe this would then visually support the result that pCO2 had an effect. Also you describe that data from the 13C treatment are missing in line 173 (which I commented on before) but why is there a data point for day 13 for the ambient pCO2 treatment but not for the elevated pCO2 treatment?

Line 254: In Figure 4 it looks like larvae used for the 18C treatment started off with smaller yolk areas already. How can this be and was the difference in yolk area size at the start accounted for when comparing the yolk area at the final day?

Discussion

Line 277-285: Can you draw some conclusion from all these Q10 values? The Q10 that you calculated is smaller than the average 2.9 calculated by Heming and Buddington. If you are using this reference, it indicates that surf smelt are less temperature dependent than most fish species studied (in case this is the point which you want to make by referring to Heming and Buddington). Why are you stating this reference? What is the point that you want to make with it? This is specifically true for the last sentence in this paragraph (line 283-285).

Line 286-292: Similar to the previous paragraph. Which point do you want to make with the review of Villalobos et al 2020? We do not know the temperature performance curve or at least the temperatures at which C. pallasi occurs in nature.

Line 310 to 322: Although there is nothing wrong with this paragraph I question why it is included. You mention that you did not measure developmental rate and you don’t know if a faster development due to higher temperature is present in surf smelt, so why do you then include this paragraph in your manuscript?

Line 337: Which temperatures are considered “temperature stress” in surf smelt larvae?

Line 338-342: What do these sentences add to your story?

Line 354-363: Again, why is this paragraph included? This paragraph is about acid-base regulation which was not part of what you have measured. You are discussing topics that you did not measure in your study.

Line 370-374: Did your embryos and larvae come from adults that were exposed to high pCO2? Relate the studies that you refer to to your own study.

Line 383-388: Also have a look at Leo et al 2018. They used herring from Norway and found higher susceptibility of embryos and larvae to high pCO2 than Sswat and Franke. But also here, the point that you want to make by referring to these herring studies is not really clear.

Line 388-390: I don’t think that you can draw this conclusion by studying surf smelt for only 3 days.

Line 415-424: While this all is correct I think it is too far off from what you did in your study and does not need to be included here.

Line 425-465: These three paragraphs are great and if they are filled out with your main points from your results they would make a great discussion making most of the previous discussion (line 264-424) redundant
