# Supplementary material for: Surf smelt accelerate usage of endogenous energy reserves under climate change
Source: PLoS One. 2022 Jun 27;17(6):e0270491. doi: 10.1371/journal.pone.0270491 (PMC9236230; doi:10.1371/journal.pone.0270491)
Supplement: Attachment — Submitted filename: Response to Reviewers.docx [file pone.0270491.s006.docx]

**Response to Reviewers**

**Editors Comments**

**Thank you for addressing concerns of the two first reviewers. Your response to my previous comments about confusing total carbon and pCO2 was not adequate, and I solicited another reviewer's opinion. You will need to re-write the manuscript with regards to pCO2 instead of total carbon in order for it to be acceptable (cf. comment #6 of Reviewer #3). Please look over the comments of reviewer #3 carefully. You should find them useful in your data analyses and discussion. Considering those points should further improve your manuscript.**

We have rewritten the manuscript focusing on pCO_2_ rather than total carbon treatments, including re-running the statistics using pCO_2_ as a continuous variable. We hope this change and modification to the subsequent manuscript fulfills this request.

**Reviewer Comments**

**1. The eggs are found buried in sediment on the beach and its unclear how much time is spent immersed in seawater. At lower tides, the eggs are in a moist environment that may heat up due to sun exposure. I imagine the CO2 levels in the sediment increase at this point relative to that found in seawater. No measurements of pH or CO2 in the sand were made. If CO2 is higher in seawater than in the emmersed sand, then one would expect high CO2 to cooccur with lower (ambient) temperatures. However, the only interaction between CO2 and temperature found was at high temperature and high CO2 (but see point 2).**

We understand that our experimental design does not replicate the dynamic nature of embryo exposure, and we acknowledge this in lines 147-151.

**2. Heart rate was highly variable but not significantly different across temperatures. The high Temp/high CO2 treatment was significantly lower but the error bars are enormous and completely overlap with the other treatments. So that result is not compelling. The text states that heart rate increased at high temp/ambient CO2, but that is not significant and so should be changed (no effect).**

When re-running the stats with pCO_2_ as a continuous variable, there was no significant effect of temperature or pCO_2_ on heartrate. The results and discussion have been modified to reflect this. The table showing experimental conditions for the heartrate experiment and the figure showing the data have been moved to supplemental materials.

**3. The text makes the case that the reduced heart rate may have resulted from CO2/pH effects on oxygen binding in the blood. However, this would only be important if the embryos were near maximum metabolic rate for the available oxygen (i.e. near the Pcrit for whatever their oxygen consumption rate was). Aerobic scope (difference between max and rest metabolic rates) is largely unknown for eggs. However, Pcrits for fish eggs below 50% saturation have been reported. In that case, any reduction in oxygen transport would not translate into a reduced metabolic rate under the oxic experimental conditions. Something to ponder.**

This possible mechanism was suggested in a source, see below. However, your points are valid and due to the unknown nature of this mechanism and wanting to avoid speculation, we have removed this sentence.

Esbaugh A (2018) Physiological implications of ocean acidification for marine fish: emerging patterns and new insights. J Comp Physiol B 188:1-13. doi:10.1007/s00360-017-1105-6

**4. Heart rate would be expected to increase with metabolic rate, which nearly always increases with temperature. An increased metabolic rate is consistent with greater consumption of yolk and fat stores at high temperature. Its unclear why heart rate wouldn’t increase at high temperature except that its so variable as to be undetectable.**

We agree and have largely removed all discussion of heart rate due, as stated above, to the fact that when we re-analyzed our data using pCO2 as a continuous variable, we did not observe a significant effect of temp or pCO2 on heart rate. And as you say, with variable data and our small sample size, it is unlikely to yield observable significant result.

**5. There is a small, but likely significant, “experiment” effect on the CO2 levels achieved (see figure below). The embryo heart rate experiment had the highest PCO2 levels achieved of any experiment. Its not possible to say whether than influenced the outcome relative to the slightly lower values in the other two experiments.**

We understand this could be an effect but as you said, it is not possible to say whether this influenced the outcome.

**6. As pointed out by the editor, the paper discusses PCO2 in the introduction and frames hypotheses about the effects of PCO2 on biology. However, the authors instead perform statistical analyses on total carbon. As stated on line 124: “While pCO2 is the parameter most commonly used to represent the suite of changes associated with OA, and is the parameter actually manipulated in many systems, CT is the control variable in this system.” It is further stated that PCO2 is temperature sensitive and this is why CT was used instead. However, both variables are temperature sensitive. In fact, what changes with temperature is the solubility of CO2. If a given amount of CO2 is dissolved in seawater, both total carbon and carbon dioxide pressure will change and this change will be different at different temperatures because of changing solubility with temperature. Cold water will hold more of the CO2 (thus higher CT) and the dissolved CO2 will exert less pressure. This is seen clearly in a graphical representation of the data (below), especially within the “ambient” treatment. So ultimately it doesn’t matter which is stated throughout the text…the treatment has high total CO2 and high PCO2 relative to the ambient control. However, because the range of values is different for each variable, the statistical analyses could be influenced. For example, if the PCO2 doubles, the pH will decrease by 0.3 units and total CO2 increases by only a small fraction because of the large amount of CO2 already dissolved in seawater. Thus analyzing the interacting effects of CT and temperature may provide less clear results than analyzing the interacting effects of PCO2 and temperature…even though they are two sides of the same coin. Because CO2 was added at a particular rate, rather than a particular amount or to achieve a particular PCO2, pH or TC, the various carbonate parameters all change with temperature in interrelated ways and the different experiments have different values.**

We agree with your summation and have re-analyzed the data using pCO2 as a continuous variable to look at pCO2 interactions. The only change to our results was the loss of statistical significance in the heartrate experiment. As a result, the manuscript has been modified to reflect these changes.
