# Supplementary material for: Surf smelt accelerate usage of endogenous energy reserves under climate change
Source: PLoS One. 2022 Jun 27;17(6):e0270491. doi: 10.1371/journal.pone.0270491 (PMC9236230; doi:10.1371/journal.pone.0270491)
Supplement: Attachment — Submitted filename: Response to Reviewers.docx [file pone.0270491.s007.docx]

**Response to Reviewers**

**1) The manuscript is still confusing pCO2 and CT. For example, in the abstract the sentence "two total carbon (CT) treatments" makes it seem like the treatments were made in a fashion other than manipulating pCO2. In fact, pCO2 was used to manipulate the system, and that should be make clear throughout the manuscript. Everyone knows that when you change pCO2, CT also changes. That sentence in the abstract should be re-written to accurately reflect the experiments.**

The CT portion of the sentence was removed from the abstract.

**2) Line 117: 20-lb**

This edit was completed.

**3) lines 133 and 134: see comment number 1 above.**

CT was changed to PCO2 when describing the manipulated variable.

**4) Lines 146 and 156: 200-mL**

This edit was completed.

**5) Lines 147-151: This sentence is awkward. Maybe break it up into 2 or three sentences?**

This sentence was broken into two sentences.

**6) Line 152: This sentence is awkward. Maybe explicitly state this is a different experiment to clarify this for the reader.**

This sentence was split and states that larval experiment was started.

**7) Line 153: 12-L**

This edit was completed.

**8) Line 167: 20-mL**

This edit was completed.

**9) Line 182: I suggest changing to ... 'into a 6-well plate labeled with its corresponding treatment.'**

This edit was completed.

**10) Please give citation for ImageJ**

A citation for ImageJ was added.

**11) Lines 189-194. This paragraph is a bit difficult to follow. I suggest re-writing it.**

This paragraph was edited for clarity.

**12) Lines 202-205 can be deleted as they appear on lines 220-222. Add the WA DFW info to the approval statement, too.**

This edit was completed.

**13) In the statistical analysis section of the methods, please state which stats software was used.**

Added in a sentence stating R software was used and added in a citation for R.

**14) Lines 225-239 and anywhere else: Please see comment number one above.**

This edit was completed.

**15) Table 3: Please correct the p for pCO2. It now reads 0.0.5418**

This edit was completed.

**16) A recent paper by Murray and Klinger (J Exp Biol (2022) 225 (5): jeb243501) is applicable to your study and could be a good addition to your discussion.**

This paper was read and added to our discussion when relevant.
